# Self-efficacy and self-regulated learning strategies as significant predictors of english writing proficiency in Chinese EFL senior high school students

**DOI:** 10.1371/journal.pone.0347121

**Published:** 2026-04-17

**Authors:** Xiaoli Chen, Jingxia Yan, Wenxiu Guo

**Affiliations:** 1 School of Education, Taiyuan Normal University, Jinzhong, Shanxi, China; 2 Foreign Languages Department, Taiyuan Normal University, Jinzhong, Shanxi, China; 3 School of Management, Shanxi Medical University, Taiyuan, Shanxi, China; Kalasalingam Academy of Research and Education, INDIA

## Abstract

Self-efficacy and self-regulated learning strategies are crucial importance for enhancing the writing proficiency of foreign language learners. This study employed a cross-sectional design to examine the relationships among writing self-efficacy, writing self-regulated learning strategies and writing proficiency in Chinese senior high school students learning English as a foreign language (EFL). A convenience sample of 223 participants completed a writing test and two adapted questionnaires: the Questionnaire of English Writing Self-Efficacy (QEWSE) and the Questionnaire of English Writing SRL Strategies (QEWSRLS). Both instruments were adapted the participants’ actual situation and showed good reliability and validity. Students’ writing proficiency was assessed based on essays scored by two independent raters. Correlation analysis was used to examine the relationships between these constructs and writing performance, and confirmatory factor analysis (CFA) was conducted to establish and validate the measurement models. The results showed significant positive relationships between the constructs, indicating that higher levels of self-efficacy and greater use of SRL strategies are associated with better writing performance. There is robust construct validity for both measurement models: a five-dimensional model of writing self-efficacy (RMSEA = 0.030) and a nine-dimensional model of writing SRL strategies (RMSEA = 0.027). These findings underscore the importance of integrating psychological and strategic factors into pedagogical practices to enhance EFL writing instruction in similar contexts.

## 1. Introduction

Writing is a complex endeavor that embodies language learners’ overall linguistic competence [1], it poses a significant challenge, particularly for learners of English as a foreign language (EFL). Its difficulty stems from its multifaceted nature, which demands the simultaneous integration of cognitive, linguistic, and metacognitive skills, often with less scaffolding than other language domains [2].

### 1.1. Literature review

From the perspective of Zimmerman, self-regulated learning (henceforth SRL) offers a critical lens to understand the processes learners employ to manage these challenges. Studies have shown that self-efficacy—an individual’s belief in their capability to execute specific tasks—plays a significant role in language learning because it influences one’s motivation and behaviors [3,4]. Although the roles of self-efficacy and SRL have been extensively established in educational research [5–7], their specific contributions to the writing proficiency of senior high school EFL learners remain inadequately explored [8,9]. Consequently, this study seeks to examine the interplay between self-efficacy, SRL strategies, and English writing proficiency among this distinct population.

Previous research on writing self-efficacy has predominantly focused on college undergraduates, where writing proficiency was typically assessed via holistic essay scoring by English professors or researchers [10,11]. A consistent finding across studies is that writing self-efficacy often emerges as a stronger predictor of writing performance than other motivational or affective factors, such as writing anxiety, particularly in regression-based models [12–14]. Furthermore, studies employing path analysis have elucidated the dual role of self-efficacy, demonstrating both its direct impact on writing outcomes and its mediating function between other variables and performance [15,16], as posited by social cognitive theory. Writing self-efficacy has also been related to a constellation of motivational variables—including writing self-concept, perceived task value, and goal orientation—and has been shown to moderate the influence of factors like gender and prior achievement [17,18]. Importantly, self-efficacy for SRL has been found to be a critical contributor not only to students’ motivational beliefs but also to their academic progress [19].

### 1.2. Theoretical framework

This study is grounded in Bandura’s social cognitive theory and Zimmerman’s cyclical phases model of SRL, with specific integration of writing self-efficacy and SRL strategies as predictors of English writing proficiency.

According to Bandura’s self-efficacy influences cognitive processes, motivation, affect, and environmental selection, operating independently of actual ability levels. Perceived self-efficacy determines learners’ effort expenditure, persistence when facing difficulties, and ultimate task performance. In writing contexts, self-efficacy shapes students’ confidence in executing specific writing tasks, thereby affecting their engagement and strategy using [5].

Zimmerman’s cyclical phases model provides the structural framework for understanding SRL strategies in writing. The model comprises three recursive phases: forethought (task analysis and self-motivational beliefs), performance (self-control and self-observation), and self-reflection (self-judgment and self-reaction). These phases emphasize the dynamic interaction among personal characteristics, behavioral processes, and environmental factors. Within this framework, SRL strategies—including goal-setting, self-monitoring, and self-evaluation—function as deliberate actions learners employ to regulate their writing processes [6,20].

Integrating these perspectives, this study posits that writing self-efficacy and SRL strategies collectively predict English writing proficiency. Self-efficacious learners are more likely to engage in forethought planning, employ performance-phase strategies, and utilize self-reflection to improve subsequent writing attempts, ultimately enhancing their writing outcomes.

### 1.3. The research questions

Although the positive effects of self-efficacy and SRL strategies on writing have been confirmed to a certain extent [4,7–10], there is still a large gap in how to systematically cultivate senior high school students’ self-efficacy and self-regulation ability and grasp their positive role in English writing learning at present. This study aims to delve into the predictive effects of various dimensions of self-efficacy and SRL on writing ability, in order to clarify the differences in the predictive weights of the five dimensions of self-efficacy and the nine dimensions of SRL strategies on writing ability. It will reveal which efficacy dimensions and strategy dimensions truly play a key role in the development of English writing for this special group of senior high school students. Providing an empirical basis for constructing targeted training programs. The research is guided by the following questions:

1. What are the underlying dimensions of writing self-efficacy and writing SRL strategies for Chinese senior high school EFL students?
2. What are the relative contributions of the self-efficacy and writing SRL strategies in predicting Chinese senior high school students’ English writing proficiency?

## 2. Materials and methods

### 2.1. Research design

This study employed a cross-sectional design with a sample of 223 participants recruited through convenience sampling. This method was chosen due to practical constraints in the educational setting, as it allowed for the efficient distribution and collection of offline questionnaires within natural classroom environments. By accessing readily available groups, sufficient data were gathered within the permitted academic schedule, ensuring the study’s practical viability. Data were collected in two phases: (1) the administration of the QEWSE and QEWSRLS questionnaires in separate 20-minute sessions, yielding 223 valid responses; and (2) a timed writing test. The essays were assessed independently by two IELTS-certified raters based on the official IELTS rubric (task response, coherence, lexicon, grammar). A final score for each essay was derived by averaging the scores across all criteria. To ensure data quality and ethical standards, rigorous protocols were implemented, including voluntary participation, minimizing participant fatigue, and maintaining assessment reliability via a double-blind scoring.

### 2.2. Participants

Participants were recruited via convenience sampling from a senior high school in a northern Chinese provincial capital. Participant recruitment starts on March 12, 2024 and ends on March 29, 2024. The survey questionnaires were mainly distributed offline, and the participants are aged 16–18.

Participation was entirely voluntary. Prior to completing the questionnaire, participants and their parents or legal guardians were informed about the purpose and procedures of the study, and parental permission together with student assent was obtained. The study protocol was approved by the Research Ethics Review Committee of Shanxi Medical University (Approval No. 2024047). All data were collected and analyzed anonymously, and no personally identifiable information was recorded.

Data collection involved a writing proficiency test and a 52-item questionnaire, which were administered to 256 students. After the removal of 33 incomplete responses, 223 datasets were retained for statistical analysis. The analyzed sample included 114 male (51.1%) and 109 female (48.9%) students, with a majority (86.1%) enrolled in the science track.

### 2.3. Instruments

#### 2.3.1. Writing test.

Participants’ English writing proficiency was assessed using an argumentative essay task from a mock university entrance examination. This simulated exam was designed to closely replicate the format, timing, and question types of the actual national test. Within the full 120-minute English paper (total score: 150), the total score for the writing section is 40 points. These 40 points are usually composed of two writing tasks, with a total word count of approximately 250 words. The first section is an application writing task, which is about 100 words. It usually requires writing an email, letter, notice, or speech, etc. The second section is reading and subsequent writing, which is about 150 words. The topic will provide an opening paragraph of a text, and the candidates are required to continue writing two more paragraphs. Their written texts are scored according to the IELTS assessment scale, which includes coherence, vocabulary use, grammatical range, and accuracy. To ensure scoring reliability, two TESOL-qualified evaluators were invited to assess the essays. Inter-rater consistency, as measured by Cronbach’s alpha, was high for both the first (α = 0.857) and the second rater (α = 0.879), with both coefficients exceeding the acceptable threshold of 0.8.

#### 2.3.2. Scales for writing self-efficacy and scales for SRL strategies for writing.

The Questionnaire of English Writing Self-Efficacy (QEWSE) was developed by adapting items from two established instruments: the Self-Efficacy for Writing Scale (SEWS) [21] and the Questionnaire of English Self-Efficacy (QESE) [22]. The QESE assesses general English self-efficacy in listening, speaking, reading, and writing. Therefore, consistent with principle of domain specificity, only the writing-related items were selected for inclusion [5].

Specifically, items from the SEWS that assessed overly basic skills (e.g., “I can punctuate my sentences correctly”) were removed. Similarly, items from the QESE that were deemed less relevant to the academic context (e.g., “I can compose messages in English on the Internet”, “I can write diaries in English”) were omitted. Furthermore, certain items were rephrased to better reflect the writing abilities of senior high school students. For instance, “I can begin my paragraph in the right spots” was adapted to “I can write a paragraph in a coherent way.” The final QEWSE comprises 23 items across five sub-scales. Respondents indicated their agreement with each statement on a 6-point Likert scale, ranging from 1 (Strongly Disagree) to 6 (Strongly Agree).

The Questionnaire of English Writing SRL Strategies (QEWSRLS) was adapted from the Questionnaire of English SRL Strategies to measure participants’ use of SRL strategies in writing [22]. The questionnaire was organized according to [23] writing self-regulation model, including three categories with 29 items: (a) environmental SRL strategies (9 items); (b) behavioral SRL strategies (9 items); and (c) personal SRL strategies (11 items). Participants were asked to rate their writing SRL strategies on a 6-point Likert scale, ranging from 1 (Strongly Disagree) to 6 (Strongly Agree). The reversed items were recorded correctly before submitting them to SPSS 27.

Before finalization, the initial item pool was refined through a two-step process. First, a panel of three EFL writing instruction experts (with over 5 years of senior high school teaching experience) and two educational measurement specialists evaluated items using three criteria: content validity, contextual relevance and age appropriateness. Then a pilot study was conducted with 30 third-year senior high school students to verify item clarity and appropriateness. Item analysis showed all items had acceptable difficulty and discrimination. Based on pilot study feedback, the item was rephrased to enhance clarity.

### 2.4. Data analysis

The data analysis proceeded in several stages using SPSS 27.0 and AMOS 29.0. Firstly, confirmatory factor analysis (CFA) was conducted to validate the measurement models. A five-factor model for writing self-efficacy (incorporating ideation, organization, grammar, English use, and distraction management) and a nine-factor model for SRL strategies (e.g., assistance-seeking, persistence, record review; opportunity-seeking, self-monitoring, self-consequences; self-evaluation, organization/transformation, goal-setting) demonstrated good fit, establishing construct validity. Subsequently, descriptive statistics and Pearson correlation analyses were performed to examine the preliminary relationships among the variables. Finally, structural equation modeling (SEM) was employed to test the hypothesized structural model. The model parameters were estimated using the maximum likelihood method, and model fit was assessed using a suite of indices, including the chi-square/degrees of freedom ratio (CMIN/DF), root mean square error of approximation (RMSEA), goodness-of-fit index (GFI), adjusted goodness-of-fit index (AGFI), normed fit index (NFI), incremental fit index (IFI), Tucker-Lewis index (TLI), and comparative fit index (CFI).

## 3. Results

### 3.1. Correlation of writing self-efficacy, writing SRL strategies and writing proficiency

**Fig 1:**
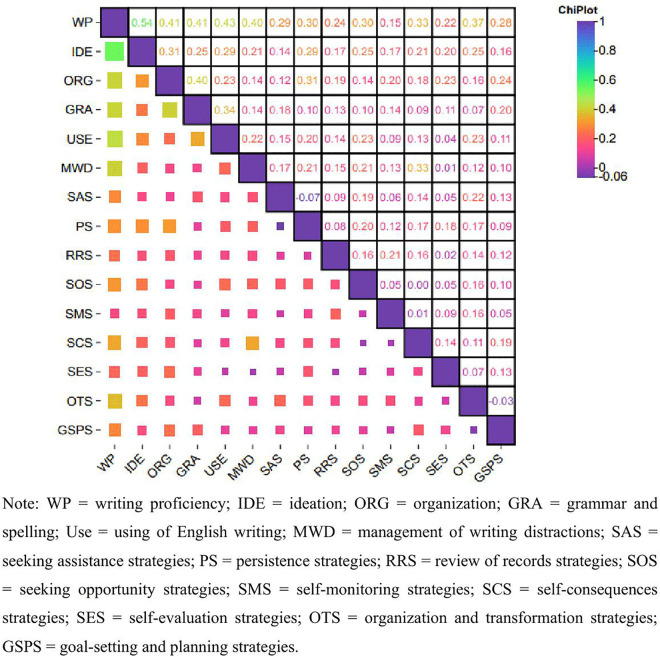
Correlation of writing self-efficacy, writing SRL strategies, and writing proficiency.

Fig 1 presents the bivariate correlations between the key constructs and writing proficiency. Among the writing self-efficacy factors, ideation demonstrated the strongest positive correlation with writing proficiency (r = 0.54, p < 0.01). All writing self-efficacy factors were significantly correlated with the outcome variable. Regarding self-regulated learning (SRL) strategies, organization and transformation strategies showed the highest correlation with writing proficiency (r = 0.37, p < 0.01). The four factors (seeking assistance strategies, persistence strategies, seeking opportunity strategies, and self-consequences strategies) indicated a moderate relationship with writing proficiency. Several other SRL strategies exhibited significant, albeit weaker, correlations with writing performance.

### 3.2. CFA of writing self-efficacy model

**Fig 2:**
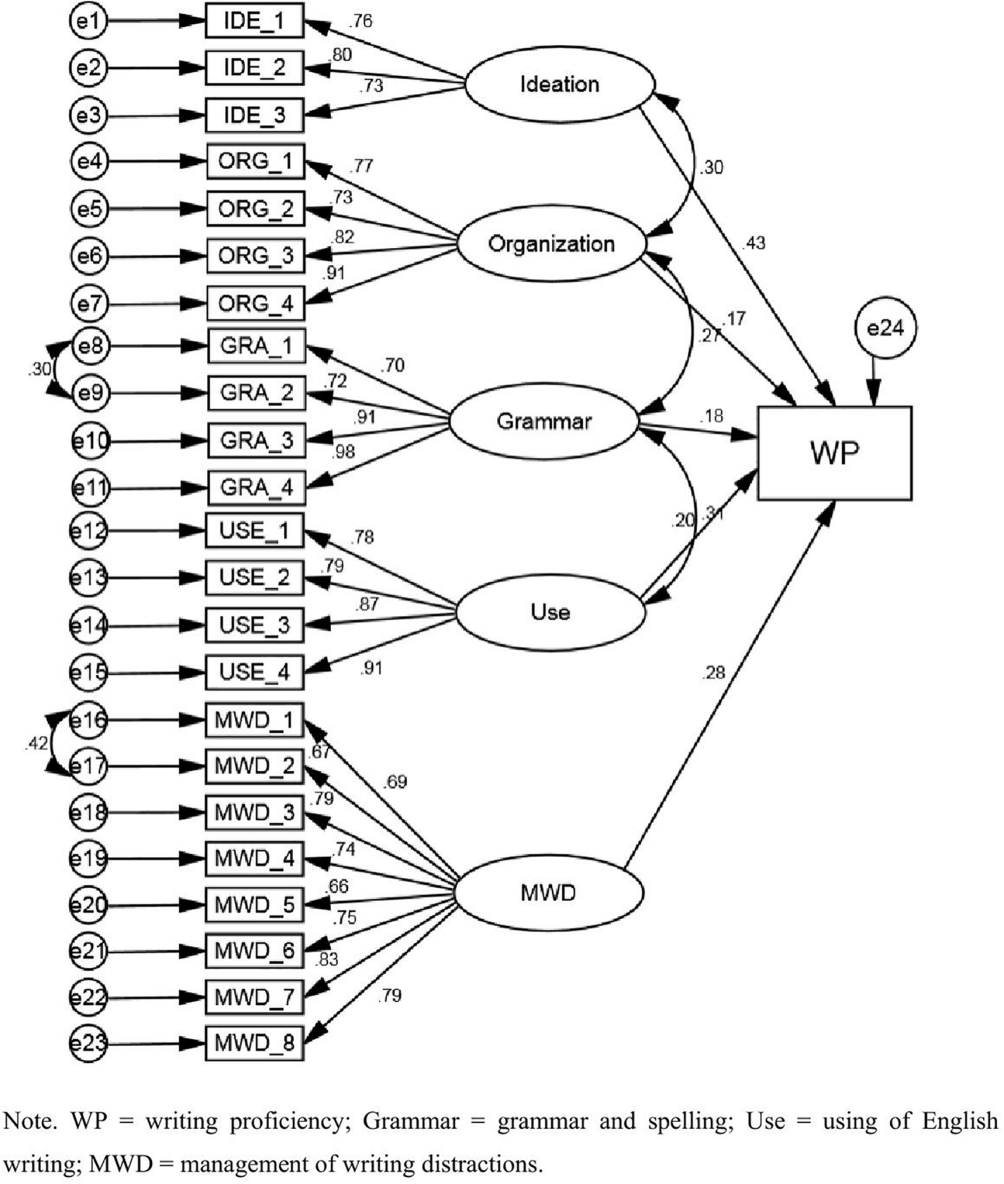
Modified CFA model for writing self-efficacy and writing proficiency.

As Table 1 showed, in the standardized regression weights of writing self-efficacy, the results of the estimates showed that there were significant positive effects on the five independent variables: ideation, organization, grammar and spelling, using of English writing, and management of writing distractions. Additionally, Fig 2 showed that ideation, using of English, and management of writing distractions were statistically significant variables in the modified model of this study (p < 0.001). Among them, ideation (β = 0.43) was considered as the most important predictor, followed by management of writing distractions (β = 0.28), using of English writing (β = 0.20), grammar and spelling (β = 0.18), and organization (β = 0.17).

**Table 1:** Modified model factor loading of writing self-efficacy and writing proficiency.

Items		Sub-Dimension	Standardized Estimate	S.E.	C.R.	P
I_1	<---	Ideation	0.755			
I_2	<---	Ideation	0.804	0.101	10.276	***
I_3	<---	Ideation	0.735	0.101	9.797	***
O_1	<---	Organization	0.766			
O_2	<---	Organization	0.730	0.085	11.097	***
O_3	<---	Organization	0.821	0.089	12.694	***
O_4	<---	Organization	0.910	0.091	13.915	***
G_1	<---	Grammar	0.700			
G_2	<---	Grammar	0.723	0.082	12.606	***
G_3	<---	Grammar	0.912	0.103	13.169	***
G_4	<---	Grammar	0.985	0.112	13.606	***
U_1	<---	Use	0.780			
U_2	<---	Use	0.791	0.081	12.635	***
U_3	<---	Use	0.868	0.078	14.159	***
U_4	<---	Use	0.915	0.084	14.952	***
S_2	<---	MWD	0.672	0.081	12.186	***
S_3	<---	MWD	0.789	0.113	10.732	***
S_4	<---	MWD	0.738	0.114	10.099	***
S_5	<---	MWD	0.659	0.107	9.096	***
S_6	<---	MWD	0.754	0.117	10.306	***
S_7	<---	MWD	0.830	0.111	11.232	***
S_1	<---	MWD	0.691			
S_8	<---	MWD	0.791	0.118	10.764	***
WP	<---	Ideation	0.433	0.038	6.388	***
WP	<---	Organization	0.170	0.035	2.719	.007
WP	<---	Grammar	0.177	0.036	2.942	.003
WP	<---	Use	0.198	0.031	3.383	***
WP	<---	MWD	0.285	0.042	4.887	***

Note. WP = writing proficiency; Grammar = grammar and spelling; Use = using of English writing; MWD = management of writing distractions.

As Table 2 showed, the goodness of fit of the modified writing self-efficacy model for CMIN/DF = 1.205, GFI = 0.902, AGFI = 0.879, NFI = 0.916, IFI = 0.985, TLI = 0.982, CFI = 0.984, and the RMSEA = 0.030, it was much less than 0.08. According to the results, only the value of AGFI did not reach 0.90, but it was very close, which was acceptable. CMIN/DF, GFI, NFI, IFI, TLI, CFI, and RMSEA met the goodness of fit.

**Table 2:** Modified CFA model fit statistics of writing self-efficacy and writing proficiency.

Indices	CMIN/DF	GFI	AGFI	NFI	IFI	TLI	CFI	RMSEA
Initial Values	1.665	0.857	0.827	0.881	0.949	0.943	0.948	0.055
Modified Values	1.205	0.902	0.879	0.916	0.985	0.982	0.984	0.030
Accepted Range	≤3.0	≥0.90	≥0.90	≥0.90	≥0.90	≥0.90	≥0.90	≤0.08

### 3.3. CFA of Writing Self-Regulated Learning Strategies Model

**Fig 3:**
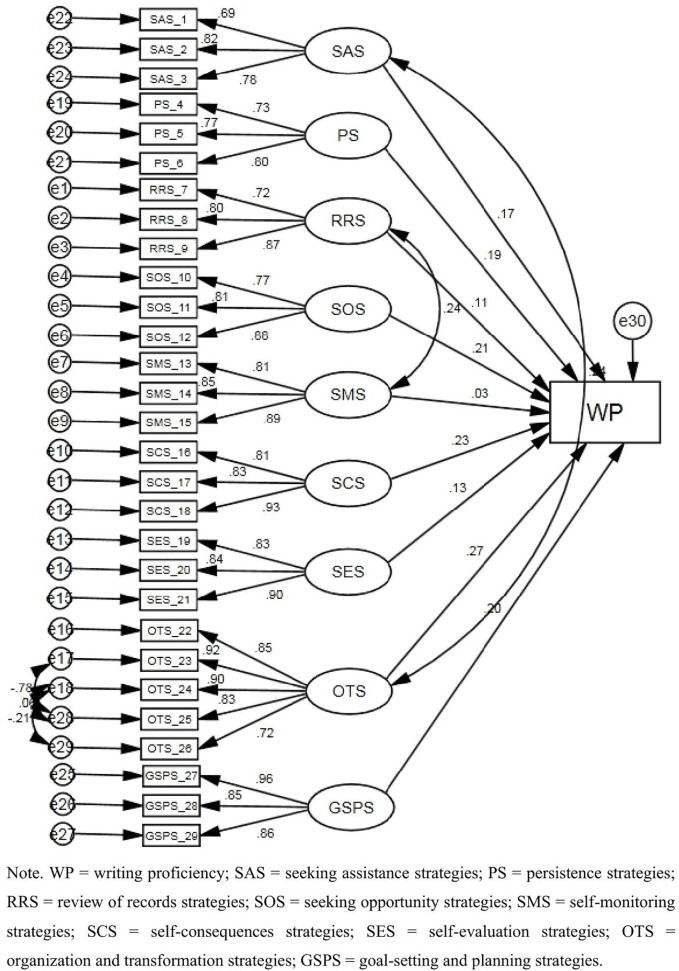
Modified CFA model of writing SRL strategies sub-dimensions and writing proficiency.

As Table 3 showed, in the standardized regression weights of writing SRL strategies, the results of the estimates showed that there were significant positive effects on the nine independent variables: seeking assistance strategies, persistence strategies, review of records strategies, seeking opportunity strategies, self-monitoring strategies, self-consequences strategies, self-evaluation strategies, organization and transformation strategies, and goal-setting and planning strategies. Additionally, Fig 3 showed that seeking opportunity strategies, self-consequences strategies, organization and transformation strategies, and goal-setting and planning strategies were statistically significant variables in the modified model of this study (p < 0.001). Among them, organization and transformation strategies (β = 0.27) were considered as the most significant predictor, followed by self-consequences strategies (β = 0.23), seeking opportunity strategies (β = 0.21), and goal-setting and planning strategies (β = 0.20).

**Table 3:** Factor loading of modified writing SRL sub-dimensions and writing proficiency model.

Items		Sub-Dimension	Standardized Estimate	S.E.	C.R.	P
RRS_7	<---	RRS	0.717			
RRS_8	<---	RRS	0.796	0.107	10.538	***
RRS_9	<---	RRS	0.870	0.116	10.647	***
SOS_10	<---	SOS	0.769			
SOS_11	<---	SOS	0.814	0.094	11.935	***
SOS_12	<---	SOS	0.875	0.107	12.233	***
SMS_13	<---	SMS	0.809			
SMS_14	<---	SMS	0.854	0.074	14.070	***
SMS_15	<---	SMS	0.894	0.073	14.457	***
SCS_16	<---	SCS	0.813			
SCS_17	<---	SCS	0.827	0.073	14.080	***
SCS_18	<---	SCS	0.933	0.076	15.238	***
SES_19	<---	SES	0.834			
SES_20	<---	SES	0.836	0.071	14.357	***
SES_21	<---	SES	0.898	0.072	15.150	***
OTS_22	<---	OTS	0.850			
OTS_23	<---	OTS	0.916	0.062	18.381	***
OTS_24	<---	OTS	0.899	0.064	17.681	***
PS_4	<---	PS	0.733			
PS_5	<---	PS	0.774	0.114	9.733	***
PS_6	<---	PS	0.804	0.113	9.788	***
SAS_1	<---	SAS	0.689			
SAS_2	<---	SAS	0.818	0.134	9.314	***
SAS_3	<---	SAS	0.780	0.138	9.290	***
GSPS_27	<---	GSPS	0.963			
GSPS_28	<---	GSPS	0.854	0.048	19.130	***
GSPS_29	<---	GSPS	0.859	0.047	19.365	***
OTS_25	<---	OTS	0.833	0.073	14.139	***
OTS_26	<---	OTS	0.718	0.064	12.780	***
WP	<---	SAS	0.168	0.044	2.548	.011
WP	<---	PS	0.187	0.037	2.929	.003
WP	<---	RRS	0.107	0.038	1.681	.093
WP	<---	SOS	0.206	0.035	3.344	***
WP	<---	SMS	0.030	0.032	.478	.633
WP	<---	SCS	0.230	0.031	3.827	***
WP	<---	SES	0.127	0.031	2.123	.034
WP	<---	OTS	0.272	0.031	4.503	***
WP	<---	GSPS	0.196	0.025	3.370	***

Note. WP = writing proficiency; SAS = seeking assistance strategies; PS = persistence strategies; RRS = review of records strategies; SOS = seeking opportunity strategies; SMS = self-monitoring strategies; SCS = self-consequences strategies; SES = self-evaluation strategies; OTS = organization and transformation strategies; GSPS = goal-setting and planning strategies.

As Table 4 showed, the goodness of fit of the modified writing self-efficacy model for CMIN/DF = 1.167, GFI = 0.880, AGFI = 0.857, NFI = 0.890, IFI = 0.983, TLI = 0.981, CFI = 0.982, and the RMSEA = 0.027, it was much less than 0.08. According to the results, the value of GFI, AGFI, and NFI did not reach 0.90, but it was very close, which was acceptable. CMIN/DF, NFI, IFI, TLI, CFI, and RMSEA met the goodness of fit.

**Table 4:** Modified CFA model fit Statistics of writing SRL strategies sub-dimensions and writing proficiency model.

Indices	CMIN/DF	GFI	AGFI	NFI	IFI	TLI	CFI	RMSEA
Initial Values	1.404	0.859	0.835	0. 867	0. 958	0. 953	0.957	0.043
Modified Values	1.167	0.880	0.857	0.890	0.983	0.981	0.982	0.027
Accepted Range	≤3.0	≥0.90	≥0.90	≥0.90	≥0.90	≥0.90	≥0.90	≤0.08

Regarding the predictive relationships, the standardized regression weights revealed that all five writing self-efficacy factors—ideation, organization, grammar and spelling, using of English writing, and management of writing distractions—had significant positive effects (p < 0.001) on the latent construct. Ideation (β = 0.433) was the strongest predictor, followed by management of writing distractions (β = 0.285), using of English writing (β = 0.198), grammar and spelling (β = 0.177), and organization (β = 0.170).

The structural equation model for writing SRL strategies was evaluated. The initial model demonstrated a reasonable fit to the data: CMIN/DF = 1.404, GFI = 0.859, AGFI = 0.835, NFI = 0.867, IFI = 0.958, TLI = 0.953, CFI = 0.957, RMSEA = 0.043. While CMIN/DF, IFI, TLI, CFI, and RMSEA indices met standard acceptability criteria, the GFI, AGFI, and NFI values fell slightly below the conventional threshold of 0.90. Model modifications were implemented to improve fit, resulting in an enhanced model with the following indices: CMIN/DF = 1.167, GFI = 0.880, AGFI = 0.857, NFI = 0.890, IFI = 0.983, TLI = 0.981, CFI = 0.982, RMSEA = 0.027. Although the GFI, AGFI, and NFI values remained slightly below 0.90, their proximity to this benchmark, combined with the excellent performance of all other indices, supported the acceptability of the final model.

Analysis of the standardized regression weights revealed that four of the nine SRL strategy factors demonstrated significant positive effects (p < 0.001) in the final model. Organization and transformation strategies (β = 0.272) emerged as the strongest predictor, followed by self-consequences strategies (β = 0.230), seeking opportunity strategies (β = 0.206), and goal-setting and planning strategies (β = 0.196).

## 4. Discussion

### 4.1. Dimensions of writing self-efficacy for writing as predictors of writing proficiency

A primary objective of this study was to identify factors influencing L2 writing proficiency by examining its relationship with writing self-efficacy among Chinese senior high school EFL students. The results demonstrated statistically significant, strong positive correlations between these constructs. Specifically, among the dimensions of writing self-efficacy, ideation exhibited the strongest association with writing proficiency, followed by management of writing distractions, use of English writing, organization, and grammar and spelling [21,24]. This pattern indicates that while overall self-efficacy is crucial, a learner’s confidence in generating and organizing ideas (ideation) is the predominant predictor of writing performance. This finding aligns with existing empirical studies [19,25], thereby reinforcing the well-established interaction between self-efficacy and writing proficiency in an EFL context.

#### 4.1.1. Ideation as a predictor.

The regression analysis confirmed that the writing self-efficacy dimensions collectively served as significant predictors of writing proficiency. Among these, ideation emerged as the strongest predictor, indicating that a learner’s confidence in generating and organizing ideas is the most critical factor influencing their writing performance [26]. This finding was consistent with a few studies that have illustrated ideation as the most important dimension in L2 writing proficiency [27]. This result can be interpreted through the writing process theory, which suggests that writers, particularly in a foreign language, often prioritize overarching meaning and ideation over lower-level concerns like grammatical accuracy during composition [28]. However, this task may have constrained the depth of ideation assessment. Thus, the strong predictive effect of ideation should be interpreted as “ability to generate concise ideas” rather than “complex argument development.” Consequently, students may develop greater self-efficacy in ideation, which in turn most directly facilitates proficient text production. To enhance writing proficiency, instruction should aim to bolster students’ ideation skills. However, the prevailing teaching paradigm in many Chinese senior high schools remains disproportionately focused on grammatical accuracy. A strategic shift towards ideation-based teaching practices—such as emphasizing brainstorming, logical structuring, and content development—is therefore crucial for aligning pedagogy with the empirical evidence on writing development.

The development of English ideation is crucial for improving L2 writing proficiency, as writing fundamentally involves externalizing ideas through acquired linguistic knowledge [29,30]. However, Chinese senior high school students often struggle with direct ideation in English, frequently reverting to L1-based thought processes—a phenomenon known as first language transfer [31]. This reliance on Chinese ideation impedes the fluency and authenticity of their English written output. Therefore, pedagogical practices should shift towards systematically cultivating students’ capacity for thinking directly in English. This can be achieved through multi-faceted instructional approaches designed to foster broad, profound, and creative ideation [32]. Specific strategies include minimizing the use of Chinese translation by providing definitions and explanations in English [33], and creating immersive classroom environments that encourage authentic English communication, thereby pushing learners to conceptualize their ideas directly in the target language [34].

#### 4.1.2. Management of writing distractions as a predictor.

This study found that the participating EFL students reported moderate overall writing self-efficacy, a finding consistent with previous researches [25,35]. A more nuanced analysis revealed that while students felt reasonably confident in managing distractions and developing ideas, their self-efficacy was notably lower in areas requiring practical application of English writing conventions, such as grammar, organization, and genre-specific composition. This pattern suggests that students perceive themselves as more capable in broader, conceptual aspects of writing but lack confidence in executing the specific linguistic and rhetorical tasks required for effective paragraph writing. This discrepancy can be interpreted through the theory of previous study [12]. The students’ lower confidence in practical writing skills likely stems from a lack of mastery experiences—the most influential source of efficacy beliefs. The EFL learning environment in this study, characterized by limited interaction with native speakers and minimal authentic writing practice (e.g., through emails or social networks), provides few opportunities for students to successfully engage in and master real-world English writing tasks. Consequently, without repeated successful experiences, their self-efficacy in applying writing conventions remains underdeveloped.

Writing self-efficacy, defined as learners’ beliefs in their capability to accomplish writing tasks, influences writing proficiency indirectly through its mediation of learning strategies. Writing self-efficacy exerts a significant influence across all three phases of self-regulated writing: forethought, performance, and self-reflection [36]. Within this framework, the “management of writing distractions” in the self-efficacy model pertains specifically to sustaining focus during the writing process, distinguishing it from the broader category of self-regulatory learning strategies. Learners with high self-efficacy are more likely to employ effective strategies, exert greater effort, and demonstrate persistence when facing challenges, independent of their actual ability level [37]. For instance, they better manage their writing time, overcome ideation obstacles, and persevere through difficulties. Furthermore, as self-efficacy strengthens, students’ self-evaluations of their writing progress become more accurate, enabling more effective self-monitoring and adjustment.

### 4.2. Dimensions of writing SRL strategies for writing as predictors of writing proficiency

This study also found a moderately significant correlation between SRL strategies and writing proficiency. Analysis revealed that writing proficiency had the highest correlation with personal SRL strategies, followed by environmental and behavioral SRL strategies. This finding is consistent with previous studies [38–40] and reveals a specific strong correlation between writing proficiency and goal-setting and planning strategies. This suggested that students who proactively establish goals and systematically plan their writing practice are more likely to achieve higher writing scores. The prominence of personal SRL strategies underscores that writing is fundamentally guided by internal processes, even as it is influenced by behavioral actions and environmental conditions [12,41,42]. For instance, a student’s success depends on their perceived competence (personal), their plan to review an assignment (behavioral), and the encouragement received from a teacher (environmental). These dimensions are interconnected; a behavioral action like finding a quiet place (environmental manipulation) is sustained only if the writer personally perceives it as effective. Specifically, within the personal SRL domain, organization/transformation strategies and goal-setting/planning strategies emerged as significant predictors of writing proficiency. This result corroborates existing literature [43–45], confirming the critical role of metacognitive management in successful writing outcomes.

#### 4.2.1. Organization and transformation strategies.

The organization and transformation of knowledge is recognized as a critical strategy in L2 writing. The findings of this study indicate that high-proficiency writers effectively organized discourse and transformed knowledge during composition, whereas low-proficiency writers predominantly relied on a “knowledge-telling” approach with minimal attempt to clarify or structure their ideas [46,47]. Furthermore, high-proficiency writers demonstrated a superior ability to accurately assess the presence of basic textual organization (e.g., introduction, main body, conclusion) in their own writing, aligning more closely with expert judgments. This suggests they possess more explicit metacognitive awareness of the structural elements incorporated into their texts. In contrast, while low-proficiency writers may monitor surface-level linguistic features, they exhibit limited capacity to evaluate the organizational coherence of their writing, even at a fundamental level. This difficulty in operating across different cognitive levels of the writing process highlights a key distinction in the strategic competence of writers at different proficiency levels [48].

The transformation strategy mentioned in this study refers to knowledge transformation and transfer during English composition, exemplified by planning a text in Chinese before writing it in English. Such practices involve the transference of conceptual and organizational schema from L1 to L2. In L2 writing research, L1 knowledge transfer is commonly categorized as positive or negative. While negative transfer may lead to errors in accuracy of vocabulary and grammar, this study supports the view that organizational and rhetorical knowledge—rooted in cognitive development rather than language-specific skills—often facilitates L2 writing. The findings indicate that students’ L1 compositional competence, particularly in textual organization, positively contributed to their English writing proficiency. This aligns with existing scholarship on the cross-linguistic applicability of higher-order writing skills. Furthermore, organization and transformation strategies were primarily employed during the writing process itself, as writers connected the topic with prior knowledge, structured their discourse, and adapted their message for the intended audience. This involved careful consideration of linguistic accuracy, content relevance, and overall coherence—demonstrating the integral role of metacognitive control in effective L2 writing.

#### 4.2.2. Goal setting and planning strategies.

Goal setting and planning strategies, whether intrinsically or extrinsically motivated, were found to be significant predictors of writing proficiency. The results suggest that students with greater awareness and engagement in goal-setting achieved higher writing scores. This aligns with the findings of which reported that a combination of moderate extrinsic and strong intrinsic goal orientation most effectively enhances student performance [49]. However, L2 writing instruction for senior high school students often remains at an emergent stage, where many learners compose texts without clear objectives or strategic planning. To address this situation, teachers should explicitly guide students in establishing purposeful writing goals, helping them recognize both the intrinsic value of self-expression and the extrinsic benefits of effective communication. Pedagogically, this involves moving beyond short-term test preparation to foster long-term development. For example, instructors can frame writing as a tool for lifelong learning and global engagement, encouraging students to see themselves as authors who exchange ideas with diverse audiences. By cultivating balanced goal orientations, educators can empower students to write with greater intention, awareness, and metacognitive control.

## 5. Conclusions and implications

### 5.1. Conclusions

This study makes a substantive contribution to L2 writing research by empirically examining the relationships among writing self-efficacy, SRL strategies, and writing proficiency in Chinese EFL context. The findings revealed that all five dimensions of writing self-efficacy served as significant predictors of writing proficiency. Ranked by the strength of their predictive effects, these dimensions were: ideation, management of writing distractions, grammar and spelling, use of English writing, and organization. This suggests that students who are confident in generating ideas, can maintain focus, and possess a solid foundation in English conventions are likely to achieve higher writing proficiency.

Furthermore, the study demonstrated that among writing SRL strategies, most sub-dimensions were positive predictors of writing performance. The most influential strategies were organization and transformation, followed by goal setting and planning, seeking assistance, seeking opportunity, self-consequences, self-evaluation, persistence, and review of records. A notable exception was the self-monitoring strategy, which exhibited a weak predictive relationship with writing proficiency, a finding that warrants further investigation.

### 5.2. Pedagogical implications

L2 writing is a complex skill that requires the integration of linguistic competence, positive psychological attributes, and strategic awareness. The findings of this study underscore the importance of learners’ ability to set and maintain appropriate goals—a key aspect of self-regulation with direct implications for senior high school English writing instruction. By guiding students to establish clear goals, refine their motivational beliefs, and monitor their writing progress, teachers can establish a virtuous cycle that promotes the sustainable development of writing proficiency. Building upon the preceding analysis of predictive factors related to self-efficacy and SRL, the following pedagogical implications are proposed.

Students’ proficiency in English writing was significantly influenced by their ideation performance, which is closely associated with stronger writing self-efficacy, clearer conceptualization, and more accurate and varied language use. Therefore, fostering Chinese senior high school students’ English writing skills requires attention to both pre-task planning and in-process composition.

Mastering English writing is a challenging and gradual process for senior high school EFL students, necessitating strategic instructional approaches to foster proficiency. To this end, a multi-faceted pedagogical framework is recommended. Instruction should adopt a progressive trajectory, moving systematically from sentence-level exercises to paragraph and ultimately full-composition tasks to build foundational skills coherently. Concurrently, diversifying writing genres and task formats is crucial for developing versatile writing competence. Furthermore, writing should not be taught in isolation but be integrated with listening, speaking, and reading activities to promote synergistic language development. During the writing process itself, providing structured prewriting guidance—such as analyzing model texts—is essential to help students internalize discourse structures and cultivate English thinking patterns, thereby minimizing L1 interference. Finally, it is imperative for both teachers and students to recognize that writing enhancement is a long-term, recursive endeavor, requiring sustained practice, motivation and a commitment to long-term development. Implementing these coordinated strategies can create a supportive ecosystem that effectively addresses the complexities of writing acquisition.

Although great care was taken when designing the research, a few limitations should be acknowledged. Firstly, the participant sample was drawn from a single senior high school, resulting in a cohort with homogeneous learning experiences, age, and proficiency levels. Consequently, the generalization of the findings to EFL populations in broader or dissimilar contexts may be limited. Secondly, the findings should be interpreted with caution due to the use of convenience sampling, which may limit the generalizability of the results to the wider population. Future research could benefit from employing probability sampling techniques to enhance representativeness. Thirdly, the exclusive reliance on quantitative methods, while revealing broader trends, may not fully capture the nuanced individual differences in learners’ perceptions of self-efficacy sources and their application of SRL strategies in specific writing tasks. To gain deeper insights, subsequent research could adopt qualitative or mixed-methods approaches, incorporating instruments such as stimulated recalls, interviews, or learning journals to triangulate data and enrich understanding.

## Supporting information

S1 Table Correlation of writing self-efficacy, writing SRL strategies and writing proficiency data. (XLSX)

S2 Table Raw data. (XLSX)

S1 File Appendix. **Research questionnaire.** This questionnaire consists of two sections: Questionnaire of English Writing Self-Efficacy (QEWSE), Questionnaire of English Writing Self-Regulated Learning Strategies (QEWSRLS).
(DOCX)
