# Supplementary material for: Self-efficacy and self-regulated learning strategies as significant predictors of english writing proficiency in Chinese EFL senior high school students
Source: PLoS One. 2026 Apr 17;21(4):e0347121. doi: 10.1371/journal.pone.0347121 (PMC13089711; doi:10.1371/journal.pone.0347121)
Supplement: S1 File — Research questionnaire. This questionnaire consists of two sections: Questionnaire of English Writing Self-Efficacy (QEWSE), Questionnaire of English Writing Self-Regulated Learning Strategies (QEWSRLS). (DOCX) [file pone.0347121.s003.docx]

**Appendix**

**Questionnaire of English Writing Self-Efficacy (QEWSE) (23 Items)**

Instruction: Please complete this questionnaire about your English writing, you may indicate①Strongly Disagree ②Disagree ③Slightly Disagree ④Slightly Agree ⑤Agree ⑥Strongly Agree. There is no wrong or right answer.

**Ideation**

1. I can think of many ideas for my English writing.

2. I can put my ideas into English writing.

3. I can focus on the main ideas when writing in English.

**Organization**

4. I can organize English sentences into a paragraph to express an idea.

5. I can organize different paragraphs into an English essay.

6. I can think of appropriate English words to describe my ideas.

7. I can write an English paragraph in a coherent way.

**Grammar and spelling**

8. I can make new English sentences with given words.

9. I can correctly use verb tenses in English writing.

10. I can write an English sentence with proper grammatical structures.

11. I can correctly spell all the words in the English essays I write.

**Use of English writing**

12. I can write an expository paragraph in English.

13. I can write email messages in English.

14. I can write a descriptive paragraph in English.

15. I can write a narrative paragraph in English.

**Management of writing distractions**

16. I can focus on my English writing for at least one hour.

17. I can finish English writing assignments in time.

18. I can plan what I want to say before I start English writing.

19. I can avoid distractions while I write in English.

20. I can revise my English writing to make it better.

21. I can control my frustration when I write in English.

22. I can keep English writing even when it's difficult.

23. I can fix my English grammar errors.

**Questionnaire of English Writing Self-Regulated Learning Strategies (QEWSRLS) (29 Items)**

Instruction: Please complete this questionnaire about your English writing, you may indicate①Strongly Disagree ②Disagree ③Slightly Disagree ④Slightly Agree ⑤Agree ⑥Strongly Agree. There is no wrong or right answer.

**Environmental SRL Strategies**

a. Seeking Assistance Strategies

1. Consult teachers when I encounter difficulties in my English writing.

2. Ask classmates when I have questions in my English writing.

3. Search related documents on internet when I have difficulties in English writing.

b. Persistence Strategies

4. Keep writing when I encounter difficulties in English writing.

5. When a friend wants to play with me, but I have not finished my writing yet, I do not play until I finish my English writing.

6. Find a quiet place to write in English when the environment is disturbing.

c. Review of Records Strategies

7. Review English texts I have learned before writing in English.

8. Review my notes of English class before writing.

9. Before I write an English essay I think about the mistakes I might make in writing.

**Behavioral SRL Strategies**

a. Seeking Opportunities Strategies

10. Use sentence patterns just learned to make new sentences for practice in English writing.

11. Try to use various English expressions to express the same meaning in writing.

12. Use words just learned to make new sentences on my initiative in English writing.

b. Self-Monitoring Strategies

13. Write down the mistakes I often make in the process of English writing.

14. Take notes in English writing classes.

15. I will reread my English essay after I finish it.

c. Self-Consequences Strategies

16. Reward myself when I make a progress in English writing.

17. Have a break when I am tired during English writing.

18. I won't get discouraged when I don't do well in writing.

**Personal SRL Strategies**

a. Self-Evaluation Strategies

19. Check my English composition before turning them in.

20. Proofread my English composition after I complete English writing.

21. When I finish my English composition, I have a rest and then read it again to check whether it should be revised.

b. Organization and Transformation Strategies

22. Write an outline before writing English essays.

23. Think out a composition in Chinese before writing it in English.

24. Make sure to write a topic sentence in each paragraph in English writing.

25. Make sure that the content of each paragraph supports its topic sentence in English writing.

26. Pay attention to the English language structure during writing.

c. Goal Setting and Planning Strategies

27. Set a goal to improve my English writing.

28. Make a plan in the process of English writing.

29. Improving my English writing skills is one of my writing goals.
